# Supplementary material for: Compartment-specific energy requirements of photosynthetic carbon metabolism in Camelina sativa leaves
Source: Planta. 2022 Apr 12;255(5):103. doi: 10.1007/s00425-022-03884-5 (PMC9005430; doi:10.1007/s00425-022-03884-5)
Supplement: Supplementary file 1 — Supplementary file1 (DOCX 44 KB) [file 425_2022_3884_MOESM1_ESM.docx]

# Compartment-specific energy requirements of photosynthetic carbon metabolism in *Camelina sativa* leaves – Supplemental information

Thomas Wieloch, Thomas David Sharkey

## Derivation of equation 1

In the absence of carbon loss due to respiration, net carbon fixation via the Calvin-Benson cycle and photorespiration, *A*, is given as

| $A=\nu_{c}-0.5\nu_{o}=V_{c}\left( 1-0.5\Phi\right)$ | (S1) |
| --- | --- |

where *ν*_c_ and *ν*_o_ denote rubisco carboxylation and oxygenation rates, respectively, and *Φ* denotes the *ν*_o_ / *ν*_c_ ratio (Farquhar et al. 1980). Solving equation S1 for *ν*_c_ yields

| $\nu_{c}=\frac{A}{1-0.5\Phi}$ | (S2) |
| --- | --- |

To calculate the rubisco carboxylation rate attributable to the CO_2_ lost in the OPPP, *ν*_c (OPPP)_, we substitute *ν*_c_ for *ν*_c (OPPP)_ and *A* for the flux through the OPPP, ν_OPPP_, which yields equation 1.

## Data tables

**Table S1** Flux and flux ratios related to rubisco and the cytosolic oxidative pentose phosphate pathway in illuminated *Camelina sativa* leaves.

|  | **Flux or** | **95% CI** | |
| --- | --- | --- | --- |
|  | **flux ratio** | **LB** | **UB** |
| ***ν*_c_** | 172.10 | 166.43 | 178.75 |
| ***ν*_o_** | 51.00 | 51.00 | 51.00 |
| ***ν*_o_ / *ν*_c_ =** *Φ* | 0.30 | 0.29 | 0.31 |
| ***ν*_OPPP_** | 6.98 | 6.92 | 7.03 |
| ***ν*_c (OPPP)_** | 8.19 | 8.07 | 8.30 |
| ***ν*_o (OPPP)_** | 2.43 | 2.30 | 2.54 |
| ***ν*_c (OPPP)_ / *ν*_c_** | 0.048 | 0.045 | 0.050 |
| ***ν*_o (OPPP)_ / *ν*_o_** | 0.048 | 0.045 | 0.050 |

Flux is given in units of μmol g^-1^ FW h^-1^. Abbreviations: *ν*_c_, rubisco carboxylation rate; *ν*_o_, rubisco oxygenation rate; *Φ*, *ν*_o_ / *ν*_c_ ratio; *ν*_OPPP_, flux through the cytosolic oxidative pentose phosphate pathway; *ν*_c (OPPP)_ and *ν*_o (OPPP)_, *ν*_c_ and *ν*_o_ accounting for the net fixation of the CO_2_ released by the cytosolic oxidative pentose phosphate pathway.

**Table S2** Metabolite and cofactor flux associated with sucrose cycling and carbon re-injection into the Calvin-Benson cycle by the cytosolic oxidative pentose phosphate pathway in illuminated *Camelina sativa* leaves [μmol g^-1^ FW h^-1^].

|  | **Metabolite** | **95% CI** | |  | **Cofactor** | **95% CI** | |
| --- | --- | --- | --- | --- | --- | --- | --- |
| **Reaction** | **flux** | **LB** | **UB** | **Cofactor** | **flux** | **LB** | **UB** |
| **Calvin-Benson cycle** | |  |  |  |  |  |  |
| PGK.p | 20.00 | 18.46 | 21.62 | **ATP** | -20.00 | -21.62 | -18.46 |
| GAPDH.p | 20.00 | 18.46 | 21.62 | **NAD(P)H** | -20.00 | -21.62 | -18.46 |
| PRK.p | 10.62 | 9.82 | 11.46 | **ATP** | -10.62 | -11.46 | -9.82 |
| **Photorespiration** | |  |  |  |  |  |  |
| GS.m | 1.21 | 1.15 | 1.27 | **ATP** | -1.21 | -1.27 | -1.15 |
| GOGAT.p | 1.21 | 1.15 | 1.27 | **Fd_red_** | -2.43 | -2.54 | -2.30 |
| GDC.m | 1.21 | 1.15 | 1.27 | **NADH** | 1.21 | 1.15 | 1.27 |
| HPR.ox | 1.21 | 1.15 | 1.27 | **NADH** | -1.21 | -1.27 | -1.15 |
| GK.p | 1.21 | 1.15 | 1.27 | **ATP** | -1.21 | -1.27 | -1.15 |
| **Oxidative pentose phosphate pathway** | | | |  |  |  |  |
| G6PD.c | 6.98 | 6.92 | 7.03 | **NADPH** | 6.98 | 6.92 | 7.03 |
| 6PGD.c | 6.98 | 6.92 | 7.03 | **NADPH** | 6.98 | 6.92 | 7.03 |
| **Sucrose cycling** | |  |  |  |  |  |  |
| UGPase.c | 2.16 | 1.84 | 2.53 | **UTP** | -2.16 | -2.53 | -1.84 |
| HK.c | 2.16 | 1.84 | 2.53 | **ATP** | -2.16 | -2.53 | -1.84 |
| FK.c | 2.16 | 1.84 | 2.53 | **ATP** | -2.16 | -2.53 | -1.84 |

Metabolite flux as reported by Xu et al. (2022). Negative and positive cofactor fluxes denote cofactor consumption and production, respectively. Intracellular location of enzyme reaction: .p, chloroplast; .m, mitochondrion; .ox, peroxisome; .c, cytosol. Enzymes: 6PGD, 6-phosphogluconate dehydrogenase; FK, fructokinase; G6PD, glucose-6-phosphate dehydrogenase; GAPDH, phosphorylating glyceraldehyde-3-phosphate dehydrogenase; GDC, glycine decarboxylase complex; GK, glycerate kinase; GOGAT, glutamine α-ketoglutarate aminotransferase; GS, glutamine synthetase; HK, hexokinase; HPR, hydroxypyruvate reductase; PGK, phosphoglycerate kinase; PRK, phosphoribulokinase; UGPase, UDP-glucose pyrophosphorylase.

**Table S3** Metabolite and cofactor flux in illuminated *Camelina sativa* leaves [μmol g^-1^ FW h^-1^].

|  | **Metabolite** | **95% CI** | |  | **Cofactor** | **95% CI** | |
| --- | --- | --- | --- | --- | --- | --- | --- |
| **Reaction** | **flux** | **LB** | **UB** | **Cofactor** | **flux** | **LB** | **UB** |
| **Calvin-Benson cycle** | |  |  |  |  |  |  |
| PGK.p | 420.20 | 408.80 | 433.42 | **ATP** | -420.20 | -433.42 | -408.80 |
| GAPDH.p | 420.20 | 408.80 | 433.42 | **NAD(P)H** | -420.20 | -433.42 | -408.80 |
| PRK.p | 223.10 | 217.43 | 229.75 | **ATP** | -223.10 | -229.75 | -217.43 |
| **Photorespiration** | |  |  |  |  |  |  |
| GS.m | 25.50 | 25.50 | 25.50 | **ATP** | -25.50 | -25.50 | -25.50 |
| GOGAT.p | 25.50 | 25.50 | 25.50 | **Fd_red_** | -51.00 | -51.00 | -51.00 |
| GDC.m | 25.49 | 25.49 | 25.49 | **NADH** | 25.49 | 25.49 | 25.49 |
| HPR.ox | 25.38 | 25.37 | 25.39 | **NADH** | -25.38 | -25.39 | -25.37 |
| GK.p | 25.38 | 25.37 | 25.39 | **ATP** | -25.38 | -25.39 | -25.37 |
| **Oxidative pentose phosphate pathway** | | | |  |  |  |  |
| G6PD.c | 6.98 | 6.92 | 7.03 | **NADPH** | 6.98 | 6.92 | 7.03 |
| 6PGD.c | 6.98 | 6.92 | 7.03 | **NADPH** | 6.98 | 6.92 | 7.03 |
| **Starch and sucrose biosynthesis, and sucrose cycling** | | | |  |  |  |  |
| AGPase.p | 10.51 | 10.51 | 10.51 | **ATP** | -10.51 | -10.51 | -10.51 |
| UGPase.c | 7.86 | 7.27 | 8.56 | **UTP** | -7.86 | -8.56 | -7.27 |
| HK.c | 2.16 | 1.84 | 2.53 | **ATP** | -2.16 | -2.53 | -1.84 |
| FK.c | 2.16 | 1.84 | 2.53 | **ATP** | -2.16 | -2.53 | -1.84 |
| **Glycolysis** | |  |  |  |  |  |  |
| PK.c | 0.99 | 0.91 | 1.08 | **ATP** | 0.99 | 0.91 | 1.08 |
| **Fatty acid biosynthesis** | | |  |  |  |  |  |
| PDC.p | 0.44 | 0.44 | 0.44 | **NADH** | 0.44 | 0.44 | 0.44 |
| ACC.p | 0.44 | 0.44 | 0.44 | **ATP** | -0.39 | -0.39 | -0.39 |
| KAR.p | 0.44 | 0.44 | 0.44 | **NADPH** | -0.39 | -0.39 | -0.39 |
| ACPr.p | 0.44 | 0.44 | 0.44 | **NADH** | -0.39 | -0.39 | -0.39 |
| **Tricarboxylic acid cycle** | | |  |  |  |  |  |
| PDC.m | 0.92 | 0.85 | 1.00 | **NADH** | 0.92 | 0.85 | 1.00 |
| IDH.m | 0.92 | 0.85 | 1.00 | **NADH** | 0.92 | 0.85 | 1.00 |
| **Amino acid biosynthesis** | | |  |  |  |  |  |
| GDH.m | 0.92 | 0.85 | 1.00 | **NADH** | -0.92 | -1.00 | -0.85 |

Metabolite flux as reported by Xu et al. (2022). Negative and positive cofactor fluxes denote cofactor consumption and production, respectively. Intracellular location of enzyme reaction: .p, chloroplast; .m, mitochondrion; .ox, peroxisome; .c, cytosol. Enzymes: 6PGD, 6-phosphogluconate dehydrogenase; ACC, acetyl-CoA carboxylase; ACPr, 2,3-trans-enoyl-ACP reductase; AGPase, ADP-glucose pyrophosphorylase; FK, fructokinase; G6PD, glucose-6-phosphate dehydrogenase; GAPDH, phosphorylating glyceraldehyde-3-phosphate dehydrogenase; GDC, glycine decarboxylase complex; GDH, glutamate dehydrogenase; GK, glycerate kinase; GOGAT, glutamine α-ketoglutarate aminotransferase; GS, glutamine synthetase; HK, hexokinase; HPR, hydroxypyruvate reductase; IDH, isocitrate dehydrogenase; KAR, 3-ketoacyl-ACP reductase; PDC, pyruvate dehydrogenase complex; PGK, phosphoglycerate kinase; PK, pyruvate kinase; PRK, phosphoribulokinase; UGPase, UDP-glucose pyrophosphorylase.

## References

Farquhar GD, Caemmerer S, Berry JA (1980) A biochemical model of photosynthetic CO_2_ assimilation in leaves of C_3_ species. Planta 149:78–90.

Xu Y, Wieloch T, Kaste JAM, et al (2022) Reimport of carbon from cytosolic and vacuolar sugar pools into the Calvin–Benson cycle explains photosynthesis labeling anomalies. PNAS 119:e2121531119.
